# Evolving Pandemic Diabetic Nephropathy

**DOI:** 10.5041/RMMJ.10005

**Published:** 2010-07-02

**Authors:** Eli A. Friedman

**Affiliations:** Distinguished Teaching Professor of Medicine, Downstate Medical Center, Brooklyn, New York, USA

**Keywords:** diabetic nephropathy, chronic kidney disease, glycation, renal failure, renoprotection

## Abstract

The expanding impact of chronic kidney disease (CKD) due to pandemic diabetes mellitus is recounted emphasizing its epidemiology that has induced global socioeconomic stress on health care systems in industrialized nations now attempting to proffer optimal therapy for end stage renal disease (ESRD). Strategies to delay and perhaps prevent progression of diabetic nephropathy from minimal proteinuria through nephrotic range proteinuria and azotemia to ESRD appear to have decreased the rate of persons with diabetes who develop ESRD. For those with ESRD attributed to diabetes, kidney transplantation affords better survival and rehabilitation than either hemodialysis or peritoneal dialysis. It is likely that advances in genetics and molecular biology will suggest early interventions that will preempt diabetic complications including renal failure.

Kidney failure in persons with diabetes has grown to be the dominant concern of the unique federal program established as a component of Medicare, that since 1972 funds maintenance hemodialysis, peritoneal dialysis, and renal transplantation for end stage renal disease (ESRD). Diabetes mellitus, initially a reason for exclusion from ESRD treatment in the 1970s, has continuously expanded to lead the list of treatable causes of irreversible renal failure to the extent that 44.6% of incident treated patients in 2006 had recognized diabetes as recorded by the United States Renal Data System (USRDS) through 2008.^1^ An additional 6.5% of patients commencing ESRD therapy had diabetes that was not noted on their Medicare Report Form. A further 10% of incident ESRD patients had diabetes diagnosed during their first year of ESRD treatment meaning that a minimum of six out of ten new ESRD patients had diabetes. ESRD and diabetes were intertwined as major drains on health resources.

The World Health Organization (WHO) predicts ongoing sharp global expansion of diabetes incidence and prevalence, estimating that by 2025 more than 300 million persons will have diabetes, raising the specter of a “Pandemic” threatening imminent collapse of socioeconomic and fiscal resources available to confront the disease onslaught. By most recent WHO estimates, while in 2000 170 million people world-wide had diabetes, by 2030 the number afflicted will reach 370 million. India and China top the “endangered” list of countries with more than 120 million persons predicted to manifest diabetes in those two countries alone. In 2005, an estimated 1.1 million people died from diabetes. WHO projects that diabetes deaths will increase by more than 50% in the next 10 years without urgent action. Most notably, diabetes deaths will increase by over 80% in upper-middle income countries between 2009 and 2019.^2^

Other than expression of alarm, there have been minimal structured efforts to prepare for a diabetes pandemic. In 2003, the author observed that: “Europe is locked in the grip of a pandemic of diabetes that now engulfs the new world.^3^ Diabetes mellitus leads the causes of ESRD in the United States, Japan, and most nations in industrialized Europe.” National ESRD registries report that both glomerulonephritis and hypertensive renal disease rank below diabetes in frequency of diagnosis among new ESRD patients, substantiating the prescient contention by Mauer and Chavers, in 1985, that “Diabetes is the most important cause of ESRD in the Western world”.^4^ Lacking any proactive plan to confront a diabetes induced ESRD pandemic, the United States and other developed nations have assumed a posture of “watchful waiting”.

Recently, however, study of the incidence curves for ESRD associated with diabetes based on data collected by the USRDS affords reason to believe that the epidemic tide is turning. Projections of unrelenting growth of diabetes and its prime complication of ESRD based on annual incident patient counts compiled by the USRDS from 1984 through 2003 were starkly frightening as shown in Figure 1. Incidence counts for the last ten years, however, show a blunting of the upward curve of incident patient counts, evident from incidence counts for 1998 through 2003 (Figure 2).

ESRD increasingly has been transformed into a geriatric disorder. As depicted in the annual data reports of the USRDS, the mean age of newly treated (incident) ESRD patients in the US has increased to above age 60 while those with diabetes are approximately two years older than those without diagnosed diabetes (Figure 4).

## NATURAL HISTORY OF DIABETIC NEPHROPATHY

Kidney disease in diabetes begins with the pathophysiologic perturbations of increased glomerular filtration rate (GFR) termed hyperfiltration, and the excretion of small amounts of albumin termed microalbuminuria (Figure 5). Thereafter, proteinuria, nephrosis, azotemia, and ESRD follow in sequence. Careful observation of the course of nephropathy in type 1 and type 2 diabetes indicates strong similarities in rate of renal functional deterioration^5^ and onset of co-morbid complications. Early nephromegaly, as well as both glomerular hyperfiltration and microalbuminuria, previously thought limited to type 1 diabetes, are now recognized as equally prevalent in type 2 diabetes.^6^ Lack of precision in diabetes classification provokes confusing terms such as “insulin requiring” to explain treatment with insulin in persons thought to have *resistant* type 2 diabetes. In fact, present criteria are unable to classify as many as one-half of diabetic persons as specifically type 1 or type 2 diabetes.^7^,^8^ Consequently, literature reports of the outcome of ESRD therapy by diabetes type are few and imprecise.

## DIABETIC COMPLICATIONS: ADVANCED GLYCOSYLATED ENDPRODUCTS (AGEs)

In health, protein alteration resulting from a non-enzymatic reaction between ambient glucose and primary amino groups on proteins to form glycated residues called Amadori products is termed the Maillard reaction. After a series of dehydration and fragmentation reactions, Amadori products are transformed to stable covalent adducts called advanced glycosylation endproducts (AGEs). In diabetes, accelerated synthesis and tissue deposition of AGEs is proposed as a contributing mechanism in the pathogenesis of clinical complications.^9^ Accumulation of AGEs in the human body progresses in aging and in complications of renal failure^10^ and diabetes.^11^ AGEs are bound to a cell surface receptor (RAGE) inducing expression of vascular cell adhesion molecule-1 (VCAM-1), an endothelial cell surface cell-cell recognition protein that can prime diabetic vasculature for enhanced interaction with circulating monocytes thereby initiating vascular injury. In addition to angiotensin-converting enzyme, chymase has been indicted as an important alternative angiotensin II-generating enzyme in hypertension and diabetes but the mechanism of chymase induction is unknown. Immunohistochemistry study of coronary and renal arteries obtained at autopsy found chymase is up-regulated in patients with diabetes along with deposition of AGEs and RAGE. It is theorized that AGEs, a hallmark of complications in diabetes, induce chymase which provokes oxidative stress via the RAGE-ERK1/2 MAP kinase pathway.^12^

The Oxidative Stress Hypothesis proposes that: hyperglycemia stimulates synthesis of oxygen free radicals that act as mediators of diabetes-associated complications. Oxidative stress is strongly implicated as a mediator of multiple diabetes-induced microvascular complications, including nephropathy, retinopathy, and distal symmetric polyneuropathy. Key mediators of glucose-induced oxidative injury are superoxide anions and nitric oxide (NO). One proposed sequence of how hyperglycemia leads to oxidative stress is that high ambient glucose levels increase mitochondrial synthesis of reactive oxygen species, activates protein kinase C (PKC) and overexpresses sorbitol. Superoxides are believed to underlie many of the oxidative changes in hyperglycemic conditions, including increases in aldose reductase and protein kinase C activity.

Mitochondrial superoxide may facilitate complications through increased synthesis of NO and, consequently, formation of the strong oxidant peroxynitrite and by poly(adenosine di-phosphate-ribose) polymerase activation.^13^ Resulting endothelial dysfunction and activation of inflammation in blood vessels drives progression of micro- and macrovasculopathy.^14^

Glomerular hyperfiltration, characteristic of the clinically silent early phase of diabetic nephropathy may be induced by Amadori protein products — in rats, infusion of glycated serum proteins induces glomerular hyperfiltration.^15^ NO, produced by endothelial cells, the most powerful vasodilator influencing glomerular hemodynamics, has enhanced activity in early experimental diabetes.^16^ Subsequently, AGEs, by quenching nitric oxide synthase activity, limit vasodilation and reduce glomerular filtration rate.^17^ Clarification of the interaction of AGEs with NO may unravel the mystery of the biphasic course of diabetic glomerulopathy — sequential hyperfiltration followed by diminished glomerular filtration.

Pharmacologic prevention of AGE formation is an attractive means of preempting diabetic microvascular complications because it bypasses the necessity of having to attain euglycemia, an often unattainable goal. Pimagidine (aminoguanidine), interferes with non-enzymatic glycosylation^18^ and reduces measured AGE levels leading to its investigation as a potential treatment. Pimagidine was selected because its structure is similar to α-hydrazinohistidine, a compound known to reduce diabetes-induced vascular leakage, while having opposite effects on histamine levels.^19^

Pimagidine treatment in rats made diabetic with streptozotocin preempts complications viewed as surrogates for human diabetic complications: 1) Preventing development of cataracts in rats 90 days after being made “moderately diabetic” (<350 mg/dL plasma glucose); lens soluble and insoluble AGE fractions were inhibited by 56% and 75% by treatment with aminoguanidine 25 mg/kg body weight starting from the day of streptozotocin injection.^20^ 2) Blocking AGE accumulation (measured by tissue fluorescence) in glomeruli and renal tubules in rats 32 weeks after induction of diabetes 32 weeks earlier; ponalrestat, an aldose reductase inhibitor, did not block AGE accumulation.^21^ Preventing glomerular basement membrane thickening typical of renal morphologic changes noted in this model of diabetic nephropathy. Blocking AGE formation to impede development of diabetic complications is an attractive strategy because of elimination of the necessity for euglycemia.^22^

Uremia in diabetes is associated with both a high serum level of AGEs and accelerated macro- and microvasculopathy. The renal clearance of AGE-peptides is 0.72 0.23 mL/min for normal subjects and 0.61 0.2 mL for diabetics with normal glomerular filtration (*P*-value NS).^23^ Diabetic uremic patients accumulate advanced glycosylated endproducts in “toxic” amounts that are not decreased to normal by hemodialysis or peritoneal dialysis^24^ but fall sharply, to within the normal range, within 8 hours of restoration of half-normal glomerular filtration by renal transplantation.^25^ A disarming, counterintuitive and unexplained report of survival in diabetic patients undergoing maintenance hemodialysis noted that there was an inverse correlation of mortality with the level of AGEs in their blood.^26^

Disappointing separate multicenter trials of aminoguanidine (Pimagidine) were conducted in adults with type 1 and type 2 diabetes and documented, fixed proteinuria of at least 500 mg/day, and a plasma creatinine concentration of <1.0 mg/dL (88 μmol/L) in women or <1.3 mg/dL (115 μmol/L) randomly assigned to treatment with aminoguanidine or placebo for four years. In the type 1 trial, 56 sites enrolled 69 subjects randomized to receive 150 or 300 mg of aminoguanidine orally b.i.d. versus placebo with a mean treatment exposure of 2.5 years. Throughout the study, more than 90% of subjects in both treatment and placebo groups were concurrently treated with either an angiotensin-converting enzyme inhibitor or receptor blocker. Compared with the placebo group, the aminoguanidine group evinced a significant (<0.05) reduction in doubling of serum creatinine concentration in those who had proteinuria >2 g/24 h. There was a non-significant “trend” toward slowing the creatinine rise in the entire group. Simultaneously, protection against diabetic retinopathy and a decrease in hyperlipidemia was noted in the treated group. Side effects in the aminoguanidine group included a transient flu-like syndrome, worsening anemia, and development of antinuclear autoantibodies (ANA).^27^ A similar study in 599 subjects with type 2 diabetes enrolled in 84 centers in Canada and the US was interrupted because of liver function abnormalities in the aminoguanidine treated group. Other adverse effects of aminoguanidine treatment included myocardial infarction, congestive heart failure, atrial fibrillation, anemia, ANA titer conversion, and upper gastrointestinal symptoms.^28^

AGE induced nephrotoxicity in diabetes has been linked to activation of protein kinase C (PKC) isoforms that promote oxidative stress. Ruboxistaurin mesylate, a bisindolylmaleimide with high specificity within the PKC gene family, inhibits PKC beta isoforms. In the streptozotocin (STZ) rat, Lepr(db)/Lepr(db) mouse, and STZ-Ren 2 rat models of diabetes, ruboxistaurin normalized glomerular hyperfiltration, decreased urinary albumin excretion, and reduced glomerular transforming growth factor-beta1 and extracellular matrix protein production.^29^ Tuttle et al. completed a promising 1 year randomized, double-blind, placebo-controlled, multicenter, pilot study in which 32 mg/day of ruboxistaurin was administered for 1 year to 123 persons with type 2 diabetes and persistent albuminuria (albumin-to-creatinine ratio (ACR) 200–2,000 mg/g), despite therapy with renin-angiotensin system inhibitors. Employing endpoints of change in ACR and estimated glomerular filtration rate (eGFR) after 1 year, urinary ACR decreased significantly (−24±9%) in participants treated with ruboxistaurin (*P* = 0.020) and non-significantly (−9±11%) in the placebo group (*P* = 0.430). Encouragingly, eGFR did not decline significantly in the ruboxistaurin group (−2.5±1.9 mL/min per 1.73 m^2^) (*P* = 0.185), but the placebo group lost significant eGFR over 1 year (−4.8±1.8 mL/min per 1.73 m^2^) (*P* = 0.009).^30^

Clinical application of aminoguanidine (2775 Library of Medicine citations as of June 2009), related molecules, or AGE breakers remains a promise unfulfilled. Lessons learned from broad investigative experience with aminoguanidine center about the species differences between induced diabetes in the rat, diabetes in the dog, and the human disease. No further human trials of aminoguanidine are planned. That AGEs and RAGE persist as a target for both prevention and amelioration of diabetic micro- and macrovascular complications is evident from current evaluations of other drugs such as TRC4186, an AGE breaker.^31^ When injected intraperitoneally at a dose of 9 or 27 mg/kg twice daily in obese Zucker spontaneously hypertensive diabetic rats, TRC4186, 27 mg/kg, prevented onset of hypertension while protecting against loss of renal function as evident from a preserved albumin to creatinine ratio and normal renal histopathology. Also disappointing, after a positive placebo controlled evaluation in a six month trial in diabetic peripheral neuropathy,^32^ ruboxistaurin had a negative placebo controlled prospective trial of its value in improving sensory nerve function in cutaneous diabetic neuropathy.^33^

## IS THE PANDEMIC OF ESRD IN THOSE WITH DIABETES SUBSIDING?

**Figure 3.**
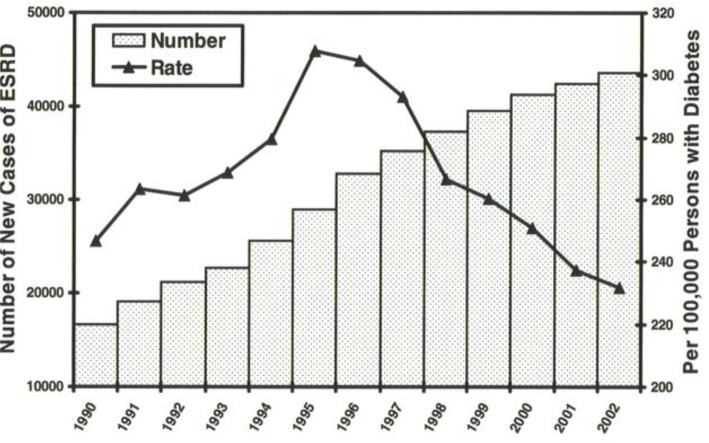
New onset end stage renal disease (ESRD) in persons with diabetes expressed as both incident number and incident rate of new onset ESRD per 100,000 persons with diabetes (United States Renal Data System (USRDS) data, 2006 (age adjusted)). A sharp decline in the incident rate starting in 1995 is evident. This observation was noted in the Centers for Disease Control and Prevention (CDC)’s Weekly Morbidity and Mortality Report in November, 2005. Inferred from this finding is the ongoing subsidence of the pandemic of ESRD in persons with diabetes.

**Figure 4.**
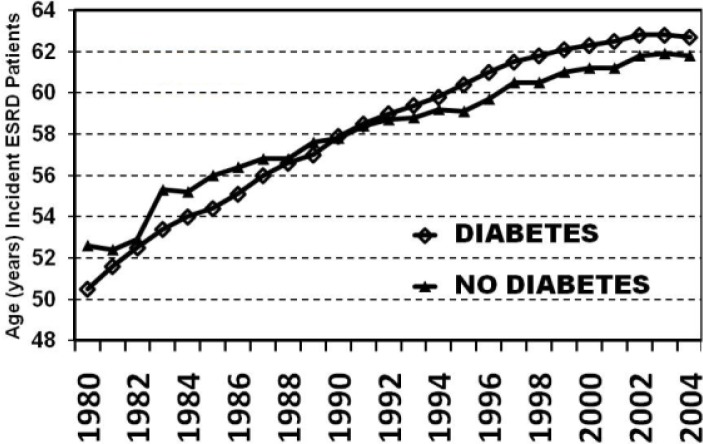
Age incident of newly treated diabetic and non-diabetic end stage renal disease (ESRD) patients in the USA between 1980 and 2004 (United States Renal Data System (USRDS) data, 2007).

**Figure 5.**
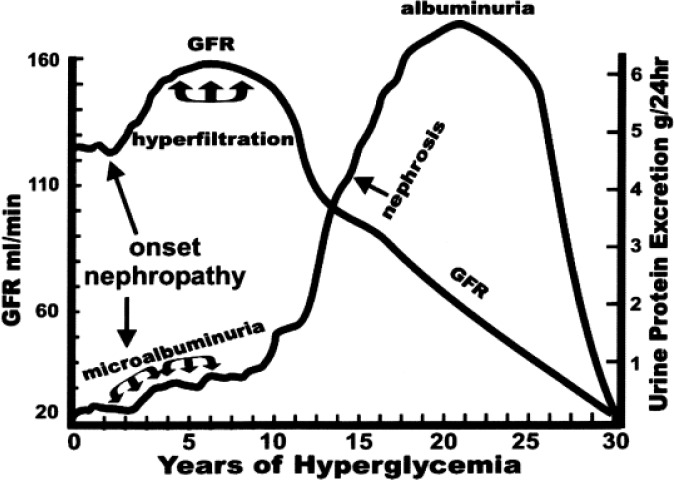
Usually first signaled by detection of small amounts (>30 mg/day) of albuminuria, the course of renal injury in individuals with diabetes is remarkably consistent and is characterized by initial nephromegaly and glomerular hyperfiltration followed by an inexorable loss of GFR accompanied by increasing proteinuria and subsequent azotemia.

First inferred from a study of ESRD incidence plotted by rate between 2000 and 2003 is the actual decline, interpreted as a major change in the epidemic “growth curve” (Figure 3), of renal failure in those with diabetes. Commenting on this “good news” presented at the end of 2005 in the Centers for Disease Control and Prevention (CDC)’s Weekly Morbidity and Mortality Report noted: “Although the number of new cases of ESRD in persons with diabetes increased overall, the incidence of ESRD-DM among persons with diabetes is not increasing among black, Hispanics, men, and persons aged 65–74 years, and is declining among persons aged <65 years, women, and whites (figures 4, 5)”.^34^ Employing as denominator all persons known to have diabetes with new incidence of ESRD as numerator revealed a remarkable sharply downward slope from a peak of 305 per 100,000 in 1996 to 232 in 2002 (*P*<0.01).

Thus, as the total United States population continues to increase, the number of diabetic persons will also increase but the proportion (rate) of diabetic individuals who will develop ESRD is falling and, by trend analysis, should continue to decrease. Why this encouraging transformation is taking place is a provocative cause for speculation. Assigning credit to the presently widely applied regimen encompassed by renoprotection is attractive.^35^ For persons with diabetes, key components of renoprotection include:

### MANAGING THE DIABETIC PATIENT

Once it is evident that the cause of renal perturbed function in a specific patient is diabetes, a strategy to prevent or delay major diabetic complications is implemented. Initially, this means establishing a program to optimize glucose control, normalize blood pressure and plasma lipids while effecting a healthy life style that includes regular exercise and a non-atherogenic diet. A key concern in constructing a treatment plan to minimize the complexity of complications in diabetes is the confusing number of drugs prescribed often at a co-payment cost that strains the patient’s budget. The subject of medication expense should be addressed repeatedly to seek lest costly substitutes.

### BLOCKING ANGIOTENSIN-CONVERTING ENZYME^36^

The adverse effect of hypertension on the course of intrinsic renal disease of any etiology is broadly appreciated. Normalizing a hypertensive blood pressure is a bedrock component of all regimens for contemporary renal care. In persons with diabetes, improvement in both the quantity of protein “leaked” into urine and the extent of normalization of hypertensive blood pressure are enhanced by treatment with an angiotensin-converting enzyme inhibitor and/or an angiotensin receptor blocker. Multiple studies and the American Diabetes Association Clinical Practice Recommendations for 2008 sustain inclusion of these drugs as first line medications in a reno-protective regimen.^37^ The natural history of diabetic nephropathy, in terms of duration of stages of chronic kidney disease (CKD) prior to onset of ESRD,^38^ has required continuous revision to reflect an improving prognosis, meaning extension of each phase of chronic kidney disease,^39^ in patients under treatment with an angiotensin-converting enzyme inhibitor (ACEi) and/or an angiotensin receptor blocker (ARB). A current advisory as to evidence based regimens holds that the renin-angiotensin-aldosterone system (RAAS) when effectively blocked by ACEi or ARB offers benefit beyond that attributable to blood pressure lowering alone by decreasing proteinuria, a risk marker for renal disease progression.^40^ Their antiproteinuric effect correlates with their kidney protection. Mounting evidence defends the hypothesis that higher doses of ACEi plus an ARB as dual RAAS blockade are more effective in reducing proteinuria increasing renoprotection.^41^

### STRIVING FOR EUGLYCEMIA^42^

Guidelines developed by the American Diabetes Association for metabolic regulation of individuals with diabetes,^43^ striving for a glycosylated hemoglobin level (HbA1c) of <7%, are not only attainable but actually cost effective for the health care system.^44^ That intensive metabolic control pays off in terms of slowing micro- and macrovasculopathy is a central tenet of current treatment strategies derived from unequivocal findings in the American Diabetes Control and Complication Trial (DCCT)^45^ and the British United Kingdom Prospective Diabetes Trial. (UKPDS).^46^ Recently, however, two large trials (ACCORD (Action to Control Cardiovascular Risk in Diabetes)^47^ and ADVANCE (Action in Diabetes and Vascular Disase: Preterax and Di-amicron Modified Release Controlled Evaluation trial)^48^) in type 2 diabetes compared the effect on cardiovascular complications of intensive and standard regimens of glucose regulation, and both indicated that near-normal glycemic control for a median of 3.5 to 5 years did not reduce cardiovascular events within that time frame. Most disturbing was the finding in the ACCORD trial that the risk of death was increased by “effective metabolic control”. The recently reported Nice-Sugar study casts further doubt on striving for strict glucose control in an intensive care setting finding that mortality at 90 days was 27.9% for strict glucose control versus 24.9% for conventional glucose control, *P*=0.02, concluding: “No additional benefit from lowering of blood glucose levels below approximately 140 to 180 mg/dL; indeed, for unclear reasons, there may be some risk”.^49^ A formal statement from in the British Medical Association advises: “The change of target A1c from 7.5% to 7% should be withdrawn before it wastes resources and possibly harms patients”.^50^

For the present, clinicians should regard the target for overall metabolic regulation in both types 1 and 2 diabetes to be a glycated hemoglobin of 7% with the understanding that while tighter control may benefit retarding of microvascular complications, the genesis of the excess deaths that prompted early termination of the ACCORD trial require further study before advocating a lower target. All other aspects of reno-protection continue as the mainstay of present diabetes care.

### CORRECTING DYSLIPIDEMIA^51^,^52^

Studies reported over the past five years link progressive nephropathy in persons with diabetes to elevated LDL cholesterol and hypertriglyceridemia as risk factors separate from proteinuria (microalbuminuria^53^). The American Diabetes Association advocates that adult patients be tested “at least annually and more often to achieve goals” of an LDL <100 mg/dL with triglycerides <150 mg/dL using statins and other lipid-lowering agents as necessary.^54^

Stopping cigarette smoking,^55^ reducing excess weight,^56^ incorporating exercise in an overall program of Life Style Modification.^57^,^58^

Whether successful alteration in life style should receive any credit for improvement in the course of those with diabetes is difficult to substantiate. Smoking cessation, weight reduction, and incorporation of foods viewed as “healthy”, are objectives more extolled than attained. Other than limited reports of low level reductions in smoking or sustained weight loss at one year, the advice though well motivated is largely hopeful rather than predictive of change. Indeed, a recent report of the proportion of persons with diabetes who actually followed advised Life Style Criteria found that only 15% of 40,000 healthy subjects participating in the National Health and Nutrition Examination Survey (NHANES 3) were adherent in 1988, of whom 8% continued their modified behavior 18 years later in 2006.^59^

Epidemiology as applied to human events is more a descriptive than a hard science. Witness our present apprehension and absence of hard information as the world prepares for what many view will be a devastating pandemic of H5N1 avian influenza.^60^,^61^ It follows that what appears to be a favorable, albeit of major medical, economic, and social impact, change in course in the epidemic curve for ESRD in diabetes must be viewed with cautious optimism rather than acceptance as fact. Nevertheless, it is inviting to ascribe benefit to a renoprotective regimen that demands so much from patients in its grasp. As documented by the USRDS, there has been a substantive increase in the proportion of “non-ESRD” diabetic individuals receiving a therapeutic regimen containing ACEi/ARB angiotensin blockade and/or lipid lowering drugs therefore meeting the definition of renoprotection.^62^ Credit for the declining incidence rate of ESRD in diabetic persons may also be due, in part, to success in the difficult to attain life style modifications of weight loss, exercise, and smoking cessation advocated by the American Diabetes Association.^63^ As noted in the Washington Post on March 9, 2006: “Americans smoked fewer cigarettes last year than at any time since 1951 … a 4.2 percent decline in 2005 alone”.^64^ Growing evidence suggests that combining an ACEi with an ARB retards progression of chronic kidney disease by optimizing reduction of proteinuria irrespective of the etiology or character of the renal disorder.^65^

## MANAGING ESRD

Choices for long-term management of irreversible uremia in diabetic patients contain the same modalities as for treating renal failure in non-diabetic patients with two additional concerns:

1. Diabetic patients generally have severe comorbidities indicating macro- and microvascular injury.
2. Pancreas and islet transplants have been successfully performed in type 1 diabetes and experimentally in selected patients with type 2 diabetes. Table 1 lists therapeutic options for diabetic ESRD patients.
  Comparison between peritoneal dialysis, hemodialysis and kidney transplantation are listed in Table 2.

## PROMISE OF PROTEOMICS

Following the previously unimaginable impact of DNA testing into the professions of criminology and security, the rapidly evolving ability to exploit minute amounts of protein in serum and urine is transforming the practice of medicine. Although discovery and quantification of micro-albuminuria is the only non-invasive current marker for diagnosing diabetic nephropathy contingent on the presence of immunoreactive forms of albumin, it may soon be possible to detect immunounreactive forms as well as other proteins that serve as biomarkers for various stages of renal injury in diabetes.^66^ Both early detection and assessment of prognosis in diabetic nephropathy appeared probable from an evaluation of urinary biomarkers in 305 individuals with diabetic and non-diabetic proteinuric renal disease employing high-resolution capillary electrophoresis coupled with electronspray ionization mass spectrometry.^67^ Among subjects with diabetes, 102 biomarkers differed significantly between those with and without nephropathy permitting 97% sensitivity and specificity in identifying the CKD as due to diabetes. An example of what to expect is afforded by a proteomic analysis of individuals at risk to type 2 diabetes discerning varying risk according to single-nucleotide polymorphisms (SNPs) in the peroxisome proliferator-activated receptor-delta gene (PPARD), i.e. rs1053049, rs6902123, and rs2267668, allowing prediction of improvement of mitochondrial function, aerobic physical fitness, and insulin sensitivity by life style intervention, as well as consequent distribution of adiposity, hepatic fat storage, and relative muscle mass.^68^

A glimpse of answers attainable via proteomic urine study was afforded by study of stored urine samples from Pima Indians with type 2 diabetes 10 years after their entry into a registry when they had no evidence of diabetic nephropathy (serum creatinine levels <1.2 mg/dL and urine albumin excretion <30 mg/g).^69^ Using surface-enhanced laser desorption/ionization time-of-flight mass spectrometry to compare 14 individuals who progressed to nephropathy with 14 who did not, 714 unique urine protein peaks were detected and organized into a 12-peak “signature” permitting correct prediction of nephropathy (89%) with 93% sensitivity.

Recently, to study the genesis of proteinuria in individuals with type 1 diabetes, the urine proteome in 12 healthy non-diabetic individuals was compared with the urine proteome in 12 subjects with type 1 diabetes and normal urinary albumin excretion rates as well as 12 subjects with type 1 diabetes and microalbuminuria.^70^ Megalin and cubilin, two multiligand receptors expressed in kidney proximal tubule cells that enable re-uptake of filtered albumin and megalin/cubilin ligands, were significantly increased in those with type 1 diabetes and microalbuminuria compared with the other two groups. Whether this finding is causative of or a response to microalbuminuria has yet to be determined. Similarly, pursuing the pathobiology of early renal changes in diabetes was explored by identifying urinary proteomes using a fluorescence-based difference gel electrophoresis and mass spectrometry techniques to identify novel biomarkers in urine samples from individuals with type 2 diabetes and normoalbuminuria, microalbuminuria, macroalbuminuria, and a control group without diabetes. E-cadherin, a specific biomarker was also studied by Western blot in urine samples and immunohistochemistry in renal biopsies. Compared with non-diabetic control subjects, urinary E-cadherin, was up-regulated 1.3-fold, 5.2-fold and 8.5-fold in those with diabetes and normoalbuminuria, microalbuminuria and macroalbuminuria respectively. The sensitivity and specificity of urinary E-cadherin for diagnosis of diabetes were 78.8% (95% CI, 74–83%) supporting the quest for urine biomarkers of clinical diagnostic value to detect the onset of diabetic nephropathy. And this is just the beginning application of what may, in the future, be termed urinomics.

## NEXT STEPS

Until the vision of curing diabetes by molecular intervention, stem cell^71^ or islet cell infusion^72^ or xenogeneic pancreas-kidney solid organ transplantation is fulfilled, arduous renoprotective regimens, described above, persists as an effective means for patients with diabetes today to raise the probability of maximizing their number of tomorrows.

## Figures and Tables

**Figure 1.**
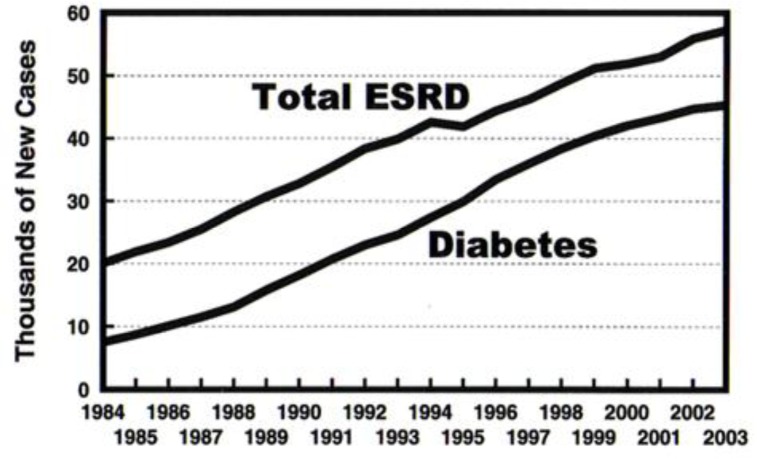
End stage renal failure incidence in the USA, compiled by the United States Renal Data System (USRDS), 2005. There has been a continuing increase in the number of new cases of ESRD between 1984 and 2003. The major diagnosis driving the upward curve is in persons with diabetes.

**Figure 2.**
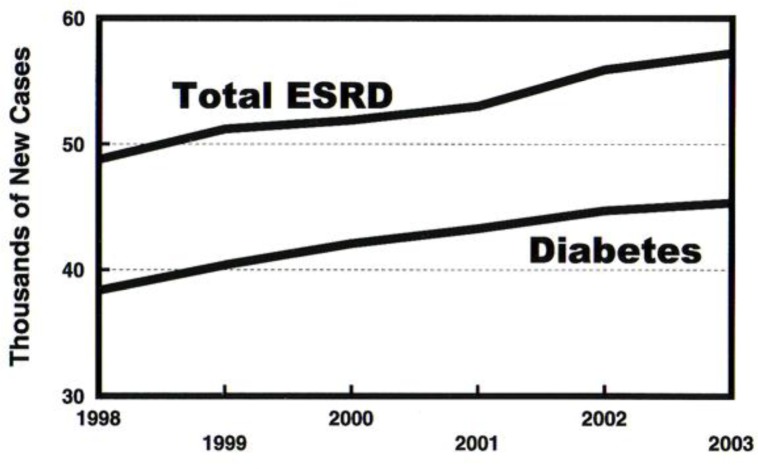
End stage renal failure incidence in the USA, compiled by the United States Renal Data System (USRDS), 2005. From 1998 to 2003, there has been a flattening of the epidemic growth curves for both diabetes and all ESRD cases. The major kidney disorder driving the upward curve is diabetes.

**Table 1.** Options in uremia therapy for diabetic ESRD patients.

1.	No specific uremia intervention = passive suicide
2.	Peritoneal dialysis
	Intermittent peritoneal dialysis (IPD)
	Continuous ambulatory peritoneal dialysis (CAPD)
	Continuous cyclic peritoneal dialysis (CCPD)
3.	Hemodialysis
	Facility hemodialysis
	Home hemodialysis
	Daily hemodialysis (nocturnal)
4.	Renal transplantation
	Deceased donor kidney
	Living donor kidney
5.	Pancreas plus kidney transplantation
	Type 1
	?Type 2 (application increasing)

**Table 2.** Composition of end stage renal disease options for diabetic patients

**Factor**	**Peritoneal Dialysis**	**Hemodialysis**	**Kidney Transplant**
Extrarenal disease	No limitation	No if hypotensive	No if severe heart disease
Geriatric patients	No limitation	No limitation	Arbitrary by program
Full rehabilitation	Rare, if ever	Rare	Common with graft functions
Death rate	Higher than non-diabetics	Higher than non-diabetics	Slightly higher than non-diabetics
First year survival	About 75–80%	About 75–80%	Above 95%
Survival >10 years	Almost never	Fewer than 5%	About one-half
Complications of diabetes	Usual plus hyperglycemia and hyperlipidemia	Usual for diabetes	Reduced by functioning transplant
Special advantage	Self-performed. No swings in blood volume level.	Can be self-performed. Efficient.	Travel freedom. Eye and nerve problems may improve
Disadvantage	Peritonitis. Long hours of treatment. More days hospitalized.	Clotting or infected access. Depression, weakness	Cosmetic disfigurement, Cost of cytotoxic drugs. Induced malignancy. HIV transmission.
Patient acceptance	Variable, usual passive tolerance for regimen.	Variable, usual passive tolerance for regimen.	Enthusiastic so long as graft functions. Exalted when pancreas normalizes glucose
Biased comparisons	First choice by enthusiasts, long-term fatigue and switch to hemodialysis.	Default for >80%. Complicated by heart and vascular disease.	Selection of healthiest and youngest patients favorably predjudices outcome.
Relative cost	First year less than kidney transplant, subsequent years more expensive.	First year less than transplant, subsequent years more expensive.	After first year, kidney transplant — alone — lowest cost option.

## Abbreviations:

ACEi: angiotensin-converting enzyme inhibitor;
ACR: albumin-to-creatinine ratio;
AGE: advanced glycosylated endproducts;
ANA: antinuclear autoantibodies;
ARB: angiotensin receptor blocker;
CKD: chronic kidney disease;
ESRD: end stage renal disease;
GFR: glomerular filtration rate;
PKC: protein kinase C;
RAGE: the receptor for advanced glycation endproducts;
USRDS: United States Renal Data System;
WHO: World Health Organization.
